# RHO binding to FAM65A regulates Golgi reorientation during cell migration

**DOI:** 10.1242/jcs.198614

**Published:** 2016-12-15

**Authors:** Faraz K. Mardakheh, Annette Self, Christopher J. Marshall

**Affiliations:** Institute of Cancer Research, Division of Cancer Biology, 237 Fulham Road, London SW3 6JB, UK

**Keywords:** Rho-GTPases, Golgi orientation, FAM65A, CCM3, MST4, Cerebral cavernous malformation

## Abstract

Directional cell migration involves reorientation of the secretory machinery. However, the molecular mechanisms that control this reorientation are not well characterised. Here, we identify a new Rho effector protein, named FAM65A, which binds to active RHOA, RHOB and RHOC. FAM65A links RHO proteins to Golgi-localising cerebral cavernous malformation-3 protein (CCM3; also known as PDCD10) and its interacting proteins mammalian STE20-like protein kinases 3 and 4 (MST3 and MST4; also known as STK24 and STK26, respectively). Binding of active RHO proteins to FAM65A does not affect the kinase activity of MSTs but results in their relocation from the Golgi in a CCM3-dependent manner. This relocation is crucial for reorientation of the Golgi towards the leading edge and subsequent directional cell migration. Our results reveal a previously unidentified pathway downstream of RHO that regulates the polarity of migrating cells through Golgi reorientation in a FAM65A-, CCM3- and MST3- and MST4-dependent manner.

## Abstract (precis)

**Highlighted Article:** Identification of a new pathway downstream of the RHO subfamily of small GTPases that regulates Golgi orientation during cell migration through FAM65A, CCM3 and the MST3 and MST4 protein kinases.

## INTRODUCTION

Establishment of front–back polarity is crucial for directional migration of mesenchymal-like cells (Etienne-Manneville, 2008). In addition to the asymmetric organisation of the cytoskeleton, which is achieved through recruitment as well as local synthesis of proteins (Mardakheh et al., 2015), polarised cells reorient their secretory traffic towards the direction of migration (Mellor, 2004). Such exocytic cargos, which originate from the Golgi, contain additional membrane, cell-surface receptors and extracellular matrix components that are required for the maintenance of the leading edge (Mellor, 2004). Crucially, reorientation of the secretory traffic is needed for polarised cell movement, suggesting that biased secretion at the front of the cell is an important component of directional migration (Yadav et al., 2009).

The exact molecular events that trigger the reorientation of the Golgi during cell migration are poorly characterised, but reorganisation of the microtubule network has been shown to be important for repositioning of the Golgi (Yadav and Linstedt, 2011). The Rho family of small GTPases are principle regulators of the cytoskeleton (Sadok and Marshall, 2014). In particular, the Rho-GTPase family member CDC42 plays a pivotal role in the regulation of polarity (Etienne-Manneville, 2004). CDC42 is activated in response to external stimuli such as chemoattractants or matrix–integrin engagement, and acts to induce cytoskeletal polarisation and Golgi reorientation through establishing the PAR6–PAR3–aPKC polarity complex (Etienne-Manneville, 2004). This complex leads to localised stabilisation of microtubule filaments (Etienne-Manneville and Hall, 2003; Etienne-Manneville et al., 2005) and engages with dynein to induce the pulling of astral microtubules (Palazzo et al., 2001), resulting in reorientation of the centrosome, which is closely associated with the Golgi. The involvement of other Rho-GTPases in the regulation of cell polarity, however, is less-well understood.

Importantly, in addition to the abovementioned cytoskeletal processes, specific signalling events within the Golgi are also required for reorientation of the secretory apparatus. For instance, ERK phosphorylation of the Golgi structural protein GRASP65, which promotes Golgi unstacking, is necessary to allow centrosome repositioning in response to polarity cues (Bisel et al., 2008). Golgi-localised activities of YSK1 and MST4 (also known as STK25 and STK26, respectively), which belong to the germinal center kinase III (GCKIII) subfamily of STE20-like protein kinases, also regulate Golgi dispersion and reorientation towards the leading edge (Preisinger et al., 2004), although the upstream regulators of these kinases in the context of Golgi reorientation have not been determined.

We here report FAM65A as a new effector for members of the RHO subfamily of Rho-GTPases. FAM65A associates with GTP-bound RHO proteins (RHOA, RHOB and RHOC) through an N-terminal HR1 domain. FAM65A also contains a C-terminal HEAT/Armadillo repeat motif (ARM) domain, which is constitutively associated with CCM3 (also known as PDCD10), MST4 and, to a lesser extent, MST3 (also known as STK24). Upon binding of RHO to FAM65A, MST4 is relocated from the Golgi to cytoplasmic punctae. This relocation is not only dependent on active RHO and FAM65A but also on CCM3. Importantly, we demonstrate that RHO-triggered relocation of MST proteins is crucial for reorientation of the Golgi and efficient directional migration. Our results reveal a new pathway downstream of RHO that controls Golgi reorientation and directional migration through regulation of MST protein localisation.

## RESULTS

### FAM65A is a new RHO effector

**Fig. 1.**
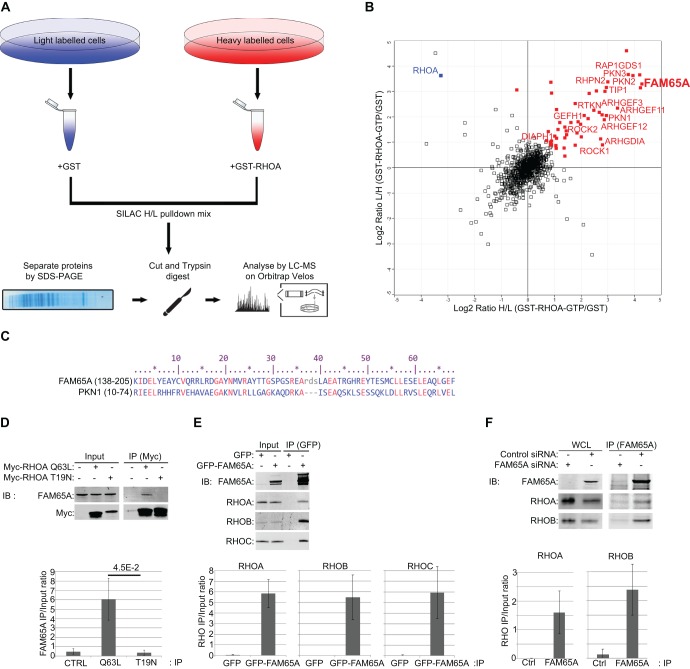
**FAM65A is a new RHO effector protein.** (A) Schematic diagram of the workflow of the quantitative-proteomics-based analysis of RHO-interacting proteins. SILAC labelled HeLa cells were lysed and subjected to affinity purification using GST–RHOA or GST-only proteins immobilised on glutathione–Sepharose beads, before in-gel digestion and mass spectrometry analysis. Heavy:light (H/L) ratios are measures of relative protein abundance between GST–RHOA and GST-only pulldowns. (B) FAM65A interacts with GST–RHOA. Log2 of SILAC ratios from two reciprocally labelled SILAC mixtures of GST–RHOA versus GST-only pulldowns (Table S1) were plotted. Proteins significantly (*P*<0.01) interacting with GST–RHOA are marked in red. Names of the known RHO-interacting proteins, as well as a new interactor named FAM65A (in bold), are depicted on the graph. The added bait RHOA protein is marked in blue. (C) The N-terminal region of FAM65A has a high sequence similarity to the N-terminal RHO-binding HR1 domain of PKN1. Identical amino acids are marked in red; similar amino acids are marked by *. (D) Endogenous FAM65A specifically interacts with active RHOA. HeLa cells were transfected with expression vectors for Myc-tagged constitutively active RHOA-Q63L, dominant-negative RHOA-T19N or empty vector as control (Ctrl), and subjected to immunoprecipitation (IP) with an anti-Myc antibody. Input lysates as well as anti-Myc immunoprecipitation eluates were subsequently analysed by immunoblotting (IB) with the indicated antibodies. Quantification of FAM65A levels in each immunoprecipitation condition relative to the input is displayed below the blots (arbitrary units). Quantifications were performed from three independent experiments. Error bars=s.d. Significance *P*-value was calculated using a two-tailed heteroscedastic *t*-test analysis. (E) All endogenous RHO proteins interacts with overexpressed FAM65A. HeLa cells were transfected with expression vectors for GFP–FAM65A or GFP-only as control, and subjected to immunoprecipitation with anti-GFP antibody. Input lysates as well as anti-GFP immunoprecipitation eluates were analysed by immunoblotting with indicated antibodies. Quantification of RHOA, RHOB and RHOC levels in each immunoprecipitation condition relative to the input is displayed below the blots (arbitrary units). Quantifications were performed from three independent experiments. Error bars=s.d. (F) Endogenous FAM65A interacts with endogenous RHOA and RHOB proteins. HeLa cells were transfected with control siRNA or siRNA against FAM65A before being subjected to immunoprecipitation with an anti-FAM65A antibody. Input lysates as well as anti-FAM65A immunoprecipitation eluates were analysed by immunoblotting with the indicated antibodies. Quantification of RHOA and RHOB levels in each immunoprecipitation condition relative to the input is displayed below the blots (arbitrary units). Quantifications were performed from three independent experiments. Error bars=s.d. WCL, whole-cell lysate.

To identify new proteins that interact with RHO, we performed pulldown assays with purified immobilised GTP-bound GST–RHOA as bait, coupled with quantitative proteomics using stable isotope labelling of amino acids in culture (SILAC) (Fig. 1A). Most known RHO effectors, as well as several upstream regulators were identified (Fig. 1B; Table S1). We also identified a number of new RHO-interacting proteins, one of which, named FAM65A, was amongst the top enriched proteins in our pulldowns (Fig. 1B; Table S1). FAM65A is an uncharacterised 132-kDa protein with no apparent catalytic domains. Amino-acid sequence analysis of FAM65A revealed the presence of a RHO-GTP-binding HR1 domain in the N-terminal region of the protein (Fig. 1C), consistent with it being a RHO effector (Flynn et al., 1998). Accordingly, FAM65A bound to a constitutively active RHOA mutant but not to a dominant-negative RHOA mutant that was defective in effector binding (Fig. 1D). Using RHO-isoform-specific antibodies (Fig. S1A), we could show that FAM65A interacts with all endogenous RHO subfamily members when overexpressed (Fig. 1E), and at least with RHOA and RHOB at endogenous levels (Fig. 1F). Collectively, these results reveal FAM65A as a new effector of the RHO subfamily of GTPases.

### FAM65A links active RHO to CCM3, MST4 and MST3

**Fig. 2.**
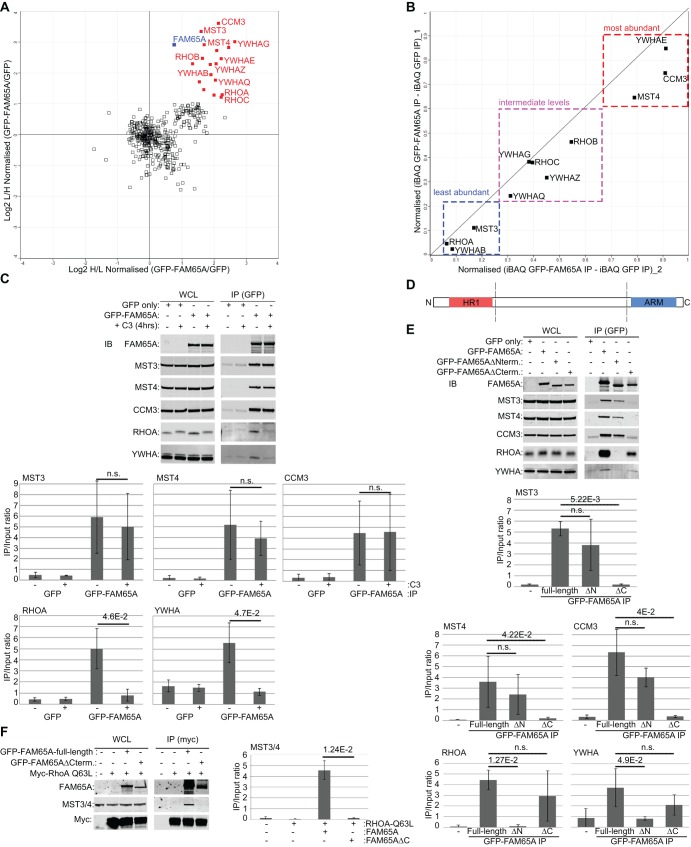
**FAM65A is an adaptor protein that links RHO to CCM3, MST3 and MST4.** (A) FAM65A interacts with all RHO proteins, as well as CCM3, MST3, MST4 and several YWHA isoforms. Averaged Log2 of SILAC ratios from replicates of two reciprocally labelled mixtures of GFP–FAM65A versus GFP-only anti-GFP immunoprecipitations (IP) (Table S2) were plotted. Proteins that significantly (*P*<0.05) interacted with GFP–FAM65A are marked in red. FAM65A, RHOA, RHOB, RHOC, CCM3, MST3, MST4 and various YWHA isoforms are depicted on the graph. FAM65A (bait) is marked in blue. H/L, heavy:light ratios. (B) Analysis of the relative stoichiometry of FAM65A-interacting proteins by iBAQ (Table S3). iBAQ values of FAM65A-interacting proteins in GFP–FAM65A immunoprecipitations were subtracted by their corresponding iBAQ values in GFP-only immunoprecipitations and normalised to FAM65A levels before being averaged between two reciprocally labelled experiments. Values from two duplicate experiments were plotted. (C) Interaction of endogenous CCM3, MST3 and MST4 with FAM65A is independent of RHO binding. HeLa cells were transfected with expression vectors for GFP–FAM65A, or GFP-only as control, and subjected to TAT-C3 or mock treatment for 4 h before lysis and immunoprecipitation with anti-GFP antibody. Input lysates as well as anti-GFP immunoprecipitation eluates were subsequently analysed by immunoblotting using the indicated antibodies. Although the RHOA and YWHA interaction with FAM65A was abrogated upon TAT-C3 treatment, interactions of CCM3, MST3 and MST4 were unaffected. Quantification of protein levels in each immunoprecipitation condition relative to the input are displayed below the blots (arbitrary units). Quantifications were performed in three independent experiments. Error bars=s.d. Significance *P*-value was calculated using two-tailed heteroscedastic *t*-test analysis. n.s., not significant (*P*>0.05). (D) Schematic representation of FAM65A regions. The N-terminal of FAM65A contains an HR1 domain (amino acids 138–205) and the C-terminal comprises an ARM domain (amino acids 1050–1202). (E) The N-terminus of FAM65A interacts with RHOA and YWHA proteins, whereas the C-terminal interacts with CCM3, MST3 and MST4. HeLa cells were transfected with expression vectors for GFP-tagged full-length, N-terminal-deleted or C-terminal-deleted FAM65A mutants, or GFP-only as control, and subjected to immunoprecipitation with anti-GFP antibody. Input lysates as well as anti-GFP immunoprecipitation eluates were subsequently analysed by immunoblotting using the indicated antibodies. Quantification of protein levels in each immunoprecipitation condition relative to the input are displayed below the blots (arbitrary units). Quantifications were performed on three independent experiments. Error bars=s.d. Significance *P*-value was calculated using two-tailed heteroscedastic *t*-test analysis. n.s., not significant (*P*>0.05). (F) FAM65A acts as an adaptor protein, linking active RHOA to MST3 and MST4. HeLa cells were co-transfected with empty vector or an expression vector for Myc–RHOA-Q63L (constitutively active), along with GFP-tagged full-length FAM65A, or the C-terminal-deleted GFP–FAM65A mutant, or GFP-only as control, and subjected to immunoprecipitation with anti-Myc antibody. Input lysates as well as anti-Myc immunoprecipitation eluates were subsequently analysed by immunoblotting using the indicated antibodies. Ectopic expression of full-length but not the C-terminal-deleted FAM65A mutant resulted in co-immunoprecipitation of endogenous MST3 and MST4 with constitutively active RHOA. Quantification of MST3 and MST4 levels in each immunoprecipitation condition relative to the input is displayed on the right-hand side of the blots (arbitrary units). Quantification was performed on three independent experiments. Error bars=s.d. Significance *P*-value was calculated using two-tailed heteroscedastic *t*-test analysis. n.s., not significant (*P*>0.05).

To reveal the proteins that interact with FAM65A in addition to the RHO subfamily members, we overexpressed GFP-tagged FAM65A in HeLa cells and performed anti-GFP immunoprecipitation coupled with quantitative proteomics analysis using SILAC (Fig. 2A; Table S2). As expected, all RHO-subfamily proteins were amongst the interactors of FAM65A. In addition, we identified CCM3, MST3 and MST4, as well as several YWHA/14-3-3 proteins amongst the specific FAM65A-interacting proteins (Fig. 2A; Table S2). MST3 and MST4 are members of the GCKIII family of kinases, which are known to bind to CCM3 (Ceccarelli et al., 2011), suggesting that the three proteins are likely to be associated with FAM65A as a complex. The third GCKIII-family member, YSK1, was not identified in FAM65A immunoprecipitations, either by using mass spectrometry (Table S2) or immunoblotting (data not shown), indicating that FAM65A selectively interacts with MST3 and MST4. Using intensity Based Absolute Quantification (iBAQ), we estimated the relative stoichiometry of the FAM65A interactions (Smits et al., 2013). YWHAE, CCM3 and MST4 were the most abundant interactors in FAM65A immunoprecipitations (Fig. 2B). MST3, however, was around fourfold-less abundant in FAM65A immunoprecipitations (Fig. 2B), indicating that the main kinase interactor of FAM65A is MST4, with MST3 associating with the complex at lower levels. Interestingly, the combined iBAQ values of MST3 and MST4 were comparable to that of CCM3, in line with the fact that CCM3 and MSTs can form a 1:1 dimer (Ceccarelli et al., 2011).

To test whether FAM65A interaction with CCM3, MST3 and MST4 was dependent on RHO, we treated GFP–FAM65A-expressing cells with a cell-permeable RHO inhibitor (TAT-C3) (Sahai and Olson, 2006) and assessed FAM65A interactions by using immunoprecipitation analyses. Although inhibition of RHO proteins resulted in their dissociation from FAM65A as expected, the CCM3, MST3 and MST4 interaction with FAM65A was unaffected by TAT-C3 treatment (Fig. 2C), suggesting that their binding to FAM65A is independent of RHO. In contrast, FAM65A interaction with YWHA/14-3-3 proteins was abrogated when RHO proteins where inhibited (Fig. 2C), suggesting that their binding to FAM65A is RHO dependent.

In addition to an N-terminal HR1 domain, amino-acid sequence analysis of FAM65A against the National Center for Biotechnology Information (NCBI) Conserved Domains Database (CDD) (Marchler-Bauer et al., 2015) suggested the presence of a C-terminal HEAT/ARM domain (Fig. 2D). ARM domains form a defined repeated structure that functions as a protein–protein interaction module (Coates, 2003). Importantly, deletion of the ARM domain abrogated FAM65A interactions with CCM3, MST3 and MST4 (Fig. 2E). In contrast, HR1-domain deletion did not abrogate FAM65A interaction with CCM3, MST3 and MST4, but abrogated RHOA binding (Fig. 2E). The binding of YWHA/14-3-3 proteins was also HR1 dependent (Fig. 2E), in line with finding that their association with FAM65A was dependent on RHO. Collectively, these results suggest that FAM65A is likely to act as an adaptor protein, linking active RHO proteins to MST3 and MST4 kinases. In support of this notion, endogenous MST3 and MST4 could be co-immunoprecipitated with a constitutively active RHOA mutant, in the presence of full-length FAM65A but not an ARM-domain-deleted mutant (Fig. 2F).

### Active RHO and FAM65A do not regulate MST activity

**Fig. 3.**
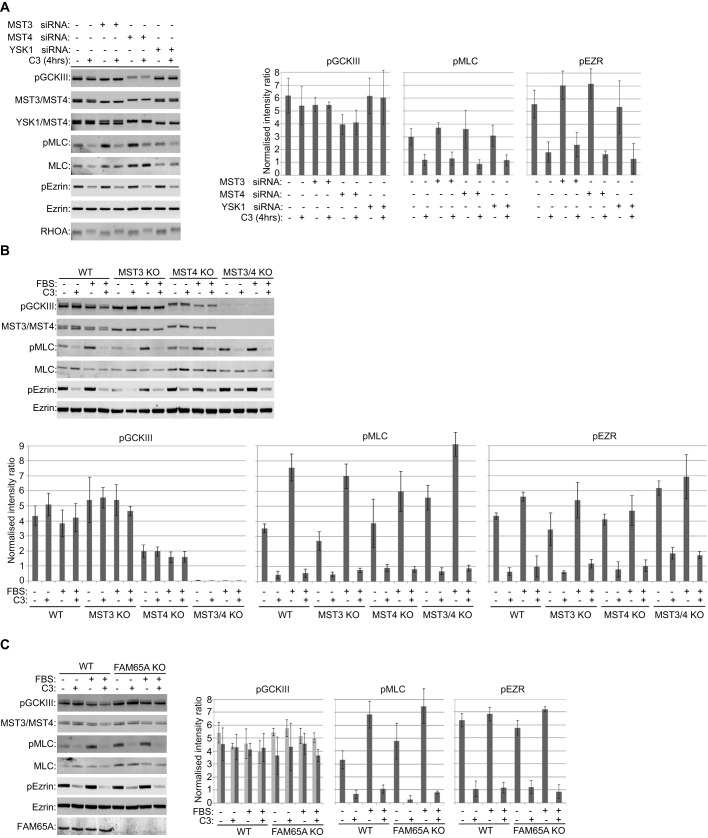
**RHO and FAM65A do not regulate MST kinase activity.** (A) The majority of GCKIII kinase activity in HeLa cells comes from MST4, with MST3 contributing to the remainder, both independently of RHO activity. HeLa cells were transfected with the indicated siRNA pools or non-targeting siRNA pool as control. 72 h post transfection, the cells were treated as indicated with TAT-C3 (C3) for 4 h, before lysis and analysis by immunoblotting with the indicated antibodies. P prefix indicates phosphorylated forms of the indicated proteins. Active phosphorylated GCKIII (pGCKIII) is resolved as a doublet, with the lower more-intense band corresponding to MST4, and the higher weaker band corresponding to MST3. TAT-C3 inactivated RHO, as manifested by a reduction in pMLC and pEzrin (pEZR) levels, but neither TAT-C3 nor YSK1 depletion affected pGCKIII levels. Bar graphs on the right-hand side of the blots display the quantifications of the indicated phosphorylated proteins. pGCKIII and pEzrin levels were normalised to total Ezrin levels as loading control, whereas pMLC was normalised to total MLC levels (arbitrary units). Quantification was performed on three independent experiments. Error bars=s.d. (B) GCKIII kinase activity comes from MST3 and MST4 and is not regulated by RHO. Wild-type (WT), MST3, MST4 or MST3 MST4 (MST3/4) double CRISPR knockout (KO) HeLa cells were starved for 24 h and treated as indicated with TAT-C3 for 4 h, before being stimulated by 10% FBS (15 min). The cells were then lysed and analysed by immunoblotting with the indicated antibodies. Stimulation activated RHO, as revealed by an increase in pMLC levels, whereas TAT-C3 inhibited RHO. Neither treatment affected pGCKIII levels, whereas double MST-knockout abrogated pGCKIII. Bar graphs below the blots display the quantifications of the indicated phosphorylated proteins. pGCKIII and pEzrin were normalised to total Ezrin levels as loading control, whereas pMLC was normalised to total MLC levels (arbitrary units). Quantification was performed on three independent experiments. Error bars=s.d. (C) GCKIII kinase activity is not regulated by FAM65A or RHO. WT or FAM65A CRISPR KO HeLa cell lines were starved for 24 h and treated as indicated with TAT-C3 for 4 h, before being stimulated by 10% FBS (15 min). The cells were then lysed and analysed by immunoblotting with the indicated antibodies. Neither FAM65A loss nor RHO activation–inactivation by FBS or TAT-C3 affected pGCKIII levels. Bar graphs on the right-hand side of the blots display the quantifications of the indicated phosphorylated proteins. pGCKIII was normalised to either total Ezrin (light grey bars) or total MST3 and MST4 (dark grey bars). pEzrin was normalised to total Ezrin, whereas pMLC was normalised to total MLC (arbitrary units). Quantification was performed on three independent experiments. Error bars=s.d.

As FAM65A provides a link between active RHO and MST proteins, we next assessed whether RHO activity can regulate MST activity in a FAM65A-dependent manner. The biochemical hallmark of the kinase activity in GCKIII kinases is phosphorylation of a conserved threonine residue within the activation segment (Thr190, Thr178 and Thr174 on MST3, MST4 and YSK1, respectively), which can be monitored by using an antibody against phosphorylated GCKIII (Gordon et al., 2011). Following RNA interference (RNAi)-mediated depletion of different GCKIII kinases, we observed that the majority of endogenous GCKIII activity corresponded to MST4, with a smaller proportion corresponding to MST3; in contrast, no proportion of the active-GCKIIII signal seem to come from YSK1 (Fig. 3A). Furthermore, CRISPR knockout of both MST3 and MST4 completely abrogated GCKIII activity (Fig. 3B), collectively suggesting that MST4 and, to a lesser extent, MST3, but not YSK1, constitute all endogenous GCKIII kinase activity. Neither serum stimulation, which activates RHO proteins, as indicated by an increase in myosin light chain (MLC, specifically MYL9, MYL12A and MYL12B) phosphorylation (Kümper et al., 2016), nor TAT-C3 treatment, which inactivates them (Sahai and Olson, 2006), affected GCKIII activity (Fig. 3A,B). Similarly, CRISPR knockout of FMA65A did not have an impact on GCKIII activity (Fig. 3C). Taken together, these results suggest that neither RHO nor FAM65A regulate the kinase activity of MST3 and MST4.

### Active RHO regulates MST4 localisation through FAM65A

**Fig. 4.**
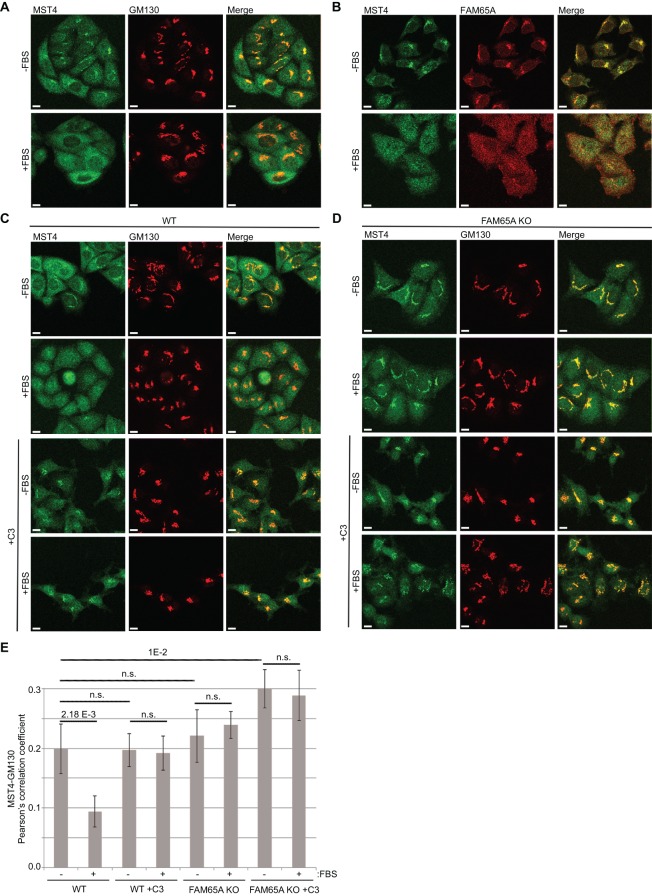
**RHO activation leads to relocation of MST4 from the Golgi to cytoplasmic punctae in a FAM65A-dependent manner.** (A) A fraction of MST4 localises to the Golgi in serum-starved cells but relocates from the Golgi upon serum stimulation. HeLa cells were seeded at low density and serum-starved for 24 h before being stimulated as indicated with 10% FBS (15 min). Cells were then fixed and immunostained with anti-GM130 (Golgi marker protein) and anti-MST4 antibodies before analysis with confocal microscopy. Scale bars: 10 µm. (B) FAM65A colocalises with MST4 in the Golgi of serum-starved cells and relocates to cytoplasmic punctae along with MST4 upon stimulation. HeLa cells were seeded at low density and serum-starved for 24 h before being stimulated as indicated with 10% FBS (15 min). Cells were then fixed and immunostained with the indicated antibodies before confocal analysis. Scale bars: 10 µm. (C) MST4 relocation from the Golgi is dependent on RHO activity. Wild-type (WT) HeLa cells were seeded at low density and serum-starved for 24 h before being treated with TAT-C3 (C3) for 4 h and stimulated with 10% FBS (15 min) as indicated. Cells were fixed and immunostained with the indicated antibodies before confocal analysis. Scale bars: 10 µm. (D) RHO-induced MST4 relocation from the Golgi is dependent on FAM65A. FAM65A CRISPR knockout (KO) HeLa cells were seeded at low density and serum-starved for 24 h before being treated with TAT-C3 for 4 h and stimulated with 10% FBS (15 min), as indicated. Cells were fixed and immunostained with the indicated antibodies before confocal analysis. Scale bars: 10 µm. (E) Quantification of colocalisation in C and D. Pearson correlation coefficients between green (MST4) and red (GM130) channels were calculated and averaged from a minimum of five independent fields of view (*n*=5) from three independent experiments, each comprised 2–12 cells per field. Error bars=s.d. Significance *P*-values were calculated using two-tailed heteroscedastic *t*-test analysis; n.s., not significant (*P*>0.05).

**Fig. 5.**
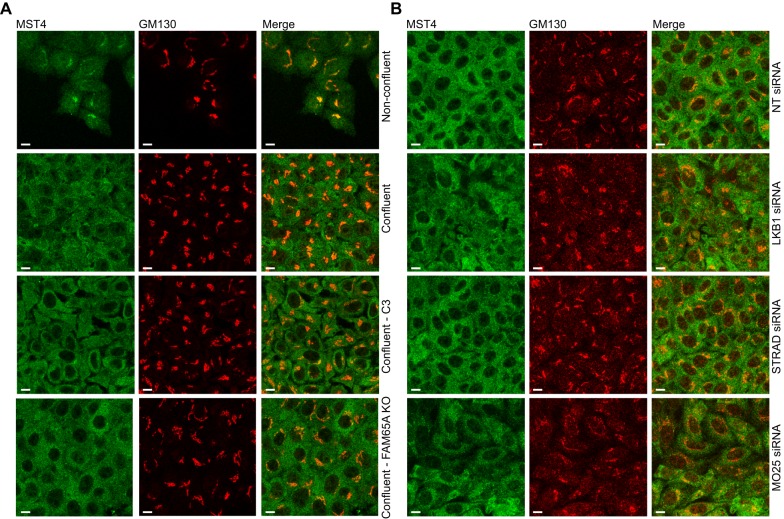
**Confluence results in relocation of MST4 from the Golgi to cytoplasmic punctae independently of RHO–FAM65A–CCM3 and LKB1–STRAD–MO25 pathways.** (A) Neither RHO inhibition by TAT-C3 nor FAM65A loss affects relocation of MST4 from the Golgi to cytoplasmic punctae in response to high cell density. Wild-type (WT) or FAM65A CRISPR knockout (KO) HeLa cells were seeded at low or high density as indicated and starved for 24 h before being treated with TAT-C3 for 4 h as shown. Cells were fixed and immunostained with anti-GM130 and anti-MST4 antibodies before confocal analysis. Scale bars: 10 µm. (B) Relocation of MST4 to cytoplasmic punctae in response to high cell density is independent of the LKB1–STRAD–MO25 polarity complex. Non-targeting (NT) control or siRNA pools against LKB1, STRAD or MO25 were transfected into HeLa cells. These cells were then seeded at high density and starved for 24 h before being fixed and immunostained with the indicated antibodies followed by confocal analysis. Scale bars: 10 µm.

Recently, it has been demonstrated that MST4 can interact with another ARM-domain-containing adaptor protein named MO25 (also known as CAB39), which is part of the polarity-regulating complex that also comprises LKB1 and STRAD (also known as STK11 and STRADA). Binding of MST4 to MO25 does not affect MST4 kinase activity but instead regulates its localisation by mediating translocation from the Golgi to the plasma membrane in response to LKB1 induction (Ten Klooster et al., 2009). We therefore hypothesised that by analogy, ARM-domain-dependent interaction of FAM65A with MST might have a role in the regulation of MST subcellular localisation downstream of active RHO. We focused on regulation of MST4 localisation because MST4 is the most active kinase in HeLa cells (Fig. 3A,B), as well as the most abundant kinase that interacts with FAM65A (Fig. 2B). We assessed the subcellular localisation of endogenous MST4 and FAM65A by performing immunofluorescence analysis using specific MST4 and FAM65A antibodies (Fig. S1B). In non-confluent serum-starved HeLa cells, a fraction of MST4 was localised to the Golgi (Fig. 4A). However, in response to serum stimulation, this MST4 relocated from the Golgi (Fig. 4A). FAM65A colocalised with the Golgi-localised MST4 in starved cells and was relocated in a similar manner upon stimulation (Fig. 4B). Higher magnification confocal analysis of serum-stimulated HeLa cells revealed that relocated MST4 and FAM65A were not diffusely distributed but instead localised to some cytoplasmic punctae (Fig. S1C). Importantly, subcellular fractionation of the starved and serum-stimulated cells revealed that the distribution of FAM65A and MST4 between the cytosol and total membrane fractions did not significantly change upon stimulation (Fig. S1D), suggesting that the cytoplasmic punctae to which both proteins relocated upon stimulation is likely to be a vesicular compartment. Crucially, inhibition of RHO activity by TAT-C3 blocked MST4 relocation (Fig. 4C,E), suggesting that this relocation depends on RHO activity. Similarly, CRISPR knockout of FAM65A abrogated MST4 relocation, with TAT-C3 having no further additive effect (Fig. 4D,E). Similar results were observed with siRNA-mediated depletion of FAM65A (Fig. S2), suggesting that the observed effects are due to the specific loss of FAM65A and not off-target effects of either CRISPR or RNAi. We also found that the Golgi localisation of MST4 was regulated by cell density, with MST4 relocating to a similar extent to that seen upon serum stimulation to cytoplasmic punctae at high confluence, but this relocation was independent of RHO activity or FAM65A (Fig. 5A). Cell-density-dependent relocation of MST4 was also independent of the LKB1–STRAD–MO25 polarity complex (Fig. 5B). These results suggest that active RHO controls MST4 localisation through FAM65A, but this regulation is dependent on cell density. We have also assessed localisation of MST3 in our system, and although we detect a small proportion of MST3 being localised to the Golgi in starved cells, the vast majority of MST3 was found outside of the Golgi (data not shown). These findings are in line with the notion that the main kinase interactor of FAM65A is MST4, with MST3 associating with FAM65A at lower levels.

### RHO regulates Golgi reorientation and cell migration through FAM65A and MST

**Fig. 6.**
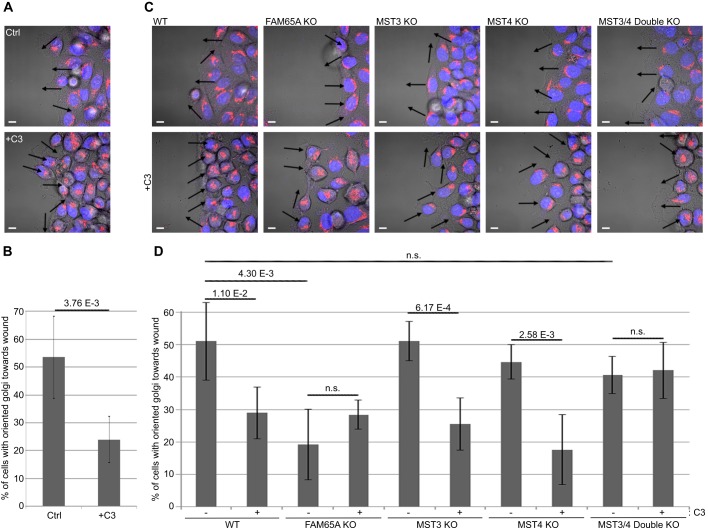
**Active RHO and FAM65A mediate Golgi reorientation by inhibiting MST3 and MST4 function.** (A) RHO inhibition impairs Golgi reorientation towards the direction of cell migration. Mock or TAT-C3 (C3)-treated migrating HeLa cells in a wound healing assay were fixed and immunostained with an anti-GM130 antibody (red) and DAPI (blue) before confocal analysis. Transmission light microscopy was used to visualise the boundaries of the cells. Scale bars: 10 µm. Arrows indicate the direction of the Golgi. (B) Quantification of Golgi orientations from A. The percentage of the edge cells with their Golgi reoriented towards the wound were calculated from five wound healing assays (*n*=5). 111–136 cells were quantified per condition. Error bars=s.d. Significance *P*-values were calculated using two-tailed heteroscedastic *t*-test analysis. (C) FAM65A loss mimics the RHO inhibition effect on Golgi reorientation, whereas double loss of MST3 and MST4 (MST3/4 KO) rescues this effect. Golgi reorientation in wild-type (WT), FAM65A, MST3, MST4 and double MST3 and MST4 CRISPR knockout (KO) HeLa cells was analysed as described in A. Scale bars: 10 µm. Arrows indicate the direction of the Golgi. (D) Quantification of Golgi orientation from C. The percentage of the edge cells with their Golgi reoriented towards the wound were calculated from five wound healing assays per condition (*n*=5). 85–153 cells were quantified per condition. Error bars=s.d. Significance *P*-values were calculated using two-tailed heteroscedastic *t*-test analysis; n.s., not significant (*P*>0.05).

It has been reported that Golgi-localised YSK1 activity is necessary for Golgi reorientation (Preisinger et al., 2004). Consequently, inhibition of YSK1 activity has been shown to block cell migration, and MST4 activity seems to oppose that of YSK1 (Preisinger et al., 2004). As active RHO regulates MST4 relocation from the Golgi through FAM65A, we hypothesised that this relocation could be important for Golgi reorientation and directional migration. To test this, we assessed Golgi reorientation in response to wounding. Three hours post wounding, over 50% of HeLa cells at the edge of the wound reoriented their Golgi towards the direction of migration. However, this was significantly impaired when RHO activity was inhibited (Fig. 6A,B). CRISPR knockout of FAM65A impaired Golgi reorientation to a similar extent, with RHO inhibition having no further additional impact on Golgi reorientation (Fig. 6C,D), suggesting that active RHO functions through FAM65A in reorienting the Golgi. In contrast, CRISPR knockout of either MST3 or MST4 did not have an impact on RHO-mediated regulation of Golgi reorientation. However, double knockout of MST3 and MST4 rendered Golgi reorientation insensitive to RHO inhibition (Fig. 6C,D). Similar results were observed with siRNA-mediated depletion of FAM65A and MST3 and MST4 (Fig. S3), together suggesting that MSTs inhibit Golgi reorientation but that this inhibition is relieved upon their relocation from the Golgi by active RHO and FAM65A. Interestingly, although MST4 is the most active GCKIII kinase and the major interactor of FAM65A, MST3 seems to be able to functionally compensate for MST4 loss given that only the combined loss of both MST3 and MST4 had an impact on the regulation of Golgi reorientation by RHO.

**Fig. 7.**
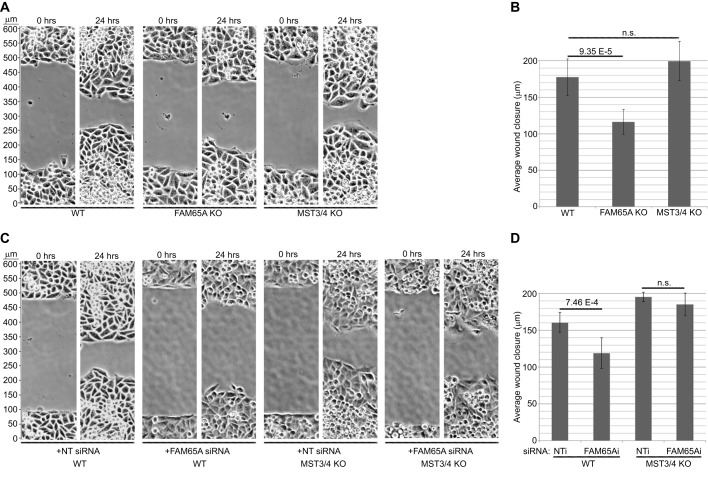
**FAM65A mediates directional migration by inhibiting MST3 and MST4 function.** (A) Loss of FAM65A impairs directional migration, whereas double loss of MST3 and MST4 does not affect directional migration. Migrating wild-type (WT), FAM65A and double MST3 and MST4 (MST3/4) CRISPR knockout (KO) HeLa cells in a wound healing assay were then analysed for 24 h by using time-lapse microscopy. Images show the wound area at indicated timepoints. (B) Quantification of the wound closure from experiments shown in A. The average distances migrated by the wound edges were calculated from three wound healing assays per condition (*n*=6). Error bars=s.d. Significance *P*-values were calculated using two-tailed heteroscedastic *t*-test analysis; n.s., not significant (*P*>0.05). (C) Impairment of directional migration upon FAM65A depletion is rescued by double loss of MST3 and MST4. Migration of WT or MST3/4 CRISPR knockout (KO) HeLa cells that had been transfected with FAM65A-specific or non-targeting (NT) siRNA pools was analysed as described in A. Images show the wound at indicated timepoints. (D) Quantification of the wound closure from the experiments shown in C. The average distances migrated by the wound edges were calculated from three wound healing assays per condition (*n*=6). Error bars=s.d. Significance *P*-values were calculated using two-tailed heteroscedastic *t*-test analysis; n.s., not significant (*P*>0.05). NTi, non targeting siRNA; FAM65Ai, siRNA targeting FAM65A.

Next, we investigated the effects of FAM65A and MST loss on the directional migration of the cells. CRISPR knockout of FAM65A impeded directional migration of HeLa cells following wounding, whereas double knockout of MST3 and MST4 did not have a significant impact on migration (Fig. 7A,B). Crucially, siRNA-mediated knockdown of FAM65A also impeded directional migration (Fig. 7C,D), but this effect could be rescued by the double CRISPR knockout of MST3 and MST4 (Fig. 7C,D). These results further support the notion that Golgi-localised MST proteins function to inhibit Golgi reorientation and directional migration, which is relieved upon their FAM65A-dependent relocation.

### CCM3 connects FAM65A to MST

**Fig. 8.**
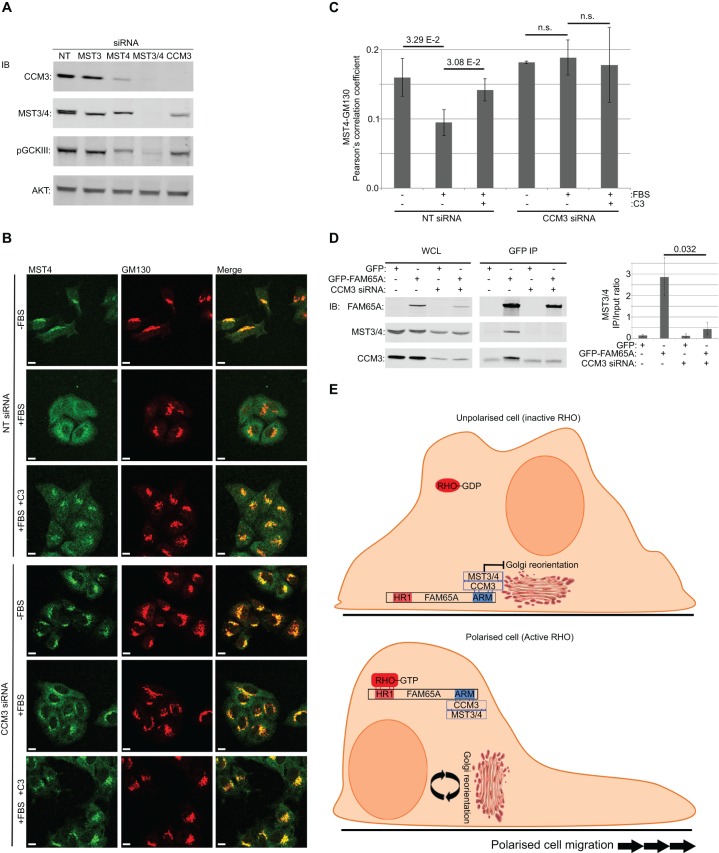
**CCM3 acts as an adaptor linking MST kinases to FAM65A.** (A) Depletion of CCM3 does not fully abrogate MST activity. HeLa cells were transfected with non-targeting control (NT) or the indicated siRNA pools. At 72 h post transfection, cells were lysed and analysed by immunoblotting with indicated antibodies. Despite a reduction in the total levels of MST3 and MST4, active phosphorylated MST kinases (pGCKIII) were still present in CCM3-depleted cells. (B) RHO-induced MST4 relocation from the Golgi is dependent on CCM3. HeLa cells that had been transfected with CCM3-specific or non-targeting control siRNA pools were seeded at low density, before being serum-starved for 24 h. Cells were treated with TAT-C3 (C3) for 4 h and stimulated with 10% FBS (15 min) as indicated, before being fixed and immunostained with the indicated antibodies for confocal analysis. Scale bars: 10 µm. (C) Quantification of colocalisation from experiments shown in B. The Pearson correlation coefficients between green (MST4) and red (GM130) channels were calculated and averaged from a minimum of three independent fields of view (*n*=3) from three experiments, each comprised 2–11 cells per field. Error bars=s.d. Significance *P*-values were calculated using two-tailed heteroscedastic *t*-test analysis; n.s., not significant (*P*>0.05). (D) Interaction of MST3 and MST4 with FAM65A is CCM3 dependent. HeLa cells were serially transfected with CCM3-specific or non-targeting control siRNA pools followed by expression vectors for GFP–FAM65A or GFP-only as control and subjected to lysis and immunoprecipitation (IP) with anti-GFP antibody. Input lysates (WCL) as well as anti-GFP immunoprecipitation eluates were analysed by immunoblotting (IB) using the indicated antibodies. Depletion of CCM3 abrogates the MST3 and MST4 (MST3/4) interaction with FAM65A. Quantification of MST3 and MST4 (MST3/4) levels in each immunoprecipitation condition relative to the input is displayed on the right-hand side of the blots (arbitrary units). Quantification was performed on three independent experiments. Error bars=s.d. Significance *P*-value was calculated using two-tailed heteroscedastic *t*-test analysis. (E) The proposed mechanism for regulation of Golgi reorientation by RHO. When RHO proteins are inactive, the FAM65A–CCM3–MST complex is localised to the Golgi, where MST proteins act to inhibit reorientation. Upon RHO activation, the FAM65A–CCM3–MST complex is relocated away from the Golgi owing to its interaction with RHO, thus relieving the inhibitory effect of MST on Golgi reorientation.

CCM3 has been shown to be important for the regulation of Golgi reorientation, and this has been shown to depend on its ability to bind to GCKIII kinases (Fidalgo et al., 2010). Binding of CCM3 to GCKIII kinases has been shown to increase their stability. Thus, it has been postulated that the function of CCM3 with regards to Golgi reorientation could be dependent on GCKIII stabilisation (Fidalgo et al., 2010). We found that although the depletion of CCM3 resulted in a decrease in MST protein levels as previously reported, this decrease was not sufficient to fully abrogate their kinase activity (Fig. 8A). However, similar to the loss of FAM65A, depletion of CCM3 inhibited relocation of MST4 from the Golgi (Fig. 8B,C), suggesting that CCM3 must also have a role in linking RHO to MST. In agreement with this notion, CCM3 depletion abrogated the interaction of endogenous MSTs with GFP–FAM65A (Fig. 8D). Together, these results suggest that the role of CCM3 is to act as an adaptor between FAM65A and MST proteins.

## DISCUSSION

Once bound to GTP, the RHO subfamily members interact with a number of effector proteins through which they can regulate several key aspects of the cytoskeletal dynamics and cell motility. These effectors include ROCK1 and ROCK2, which induce actomyosin contractility (Kümper et al., 2016; Sadok et al., 2015), the Diaphanous-related formin protein-1 (DIAPH1), which mediate actin polymerisation, and protein kinases N-1, -2 and -3 (PKN1, PKN2 and PKN3), which have been shown to regulate a host of cytoskeletal and endocytic trafficking processes (Thumkeo et al., 2013). Here, we report that RHO proteins also regulate cell polarity and directional migration by triggering Golgi reorientation through FAM65A. We demonstrate that FAM65A is an adaptor protein comprising an N-terminal HR1 domain and a C-terminal ARM domain. The HR1 domain interacts with RHO proteins in a GTP-dependent manner, whereas the ARM domain constitutively binds to CCM3, which in turn associates with MST3 and MST4. Importantly, we show that MST4 is the most important kinase that interacts with FAM65A as well as the most active GCKIII kinase in our system, but MST3 can functionally compensate for MST4 upon its loss. FAM65A and RHO activity do not have an impact on MST3 and MST4 kinase activity, but active RHO binding to FAM65A results in relocation of MST4 from the Golgi to some cytoplasmic punctae, in a FAM65A- and CCM3-dependent manner. Because RHO proteins are membrane-anchored through isoprenylation (Ridley, 2013), these cytoplasmic punctae are likely to be active RHO-containing vesicular compartments. Based on our results, we propose that the Golgi-localised MST kinases function as inhibitors of Golgi reorientation. Upon RHO activation, RHO–GTP binds to FAM65A and relocates the FAM65A–CCM3–MST complex from the Golgi, thus relieving the inhibitory effects of MSTs on Golgi reorientation (Fig. 8E).

As mentioned above, a parallel pathway has been described in gut epithelial cells in which another ARM-domain-containing protein, MO25, interacts with MST4 and mediates its relocation from the Golgi to the plasma membrane (Ten Klooster et al., 2009). This relocation is triggered by induction of the polarity master regulator LKB1 and results in phosphorylation of Ezrin by MST4 at the plasma membrane in a RAP2A-GTPase-dependent manner (Ten Klooster et al., 2009; Gloerich et al., 2012). It is, however, unclear whether the relocation of MST4 from the Golgi through MO25 also regulates Golgi reorientation. Moreover, although Ezrin phosphorylation is well known to be regulated by RHO (Shaw et al., 1998; Matsui et al., 1999), neither FAM65A nor MSTs were involved in mediating Ezrin phosphorylation in our system (Fig. 3A–C). Thus, in spite of the similarities between FAM65A- and MO25-dependent MST4 relocation events, these proteins seem to serve in functionally distinct processes. We found that confluence can also regulate MST localisation, but this regulation was independent of both RHO–FAM65A and LKB1–STRAD–MO25 pathways (Fig. 5A,B), suggesting that an as yet unknown third pathway must exist, which specifically regulates MST localisation in response to cell density.

The Golgi is generally in close association with the centrosome through the cis-Golgi-resident protein GMAP210, which directly binds to γ-tubulin (Infante et al., 1999), and reorientation of the Golgi is known to be regulated through reorganisation of the centrosome-connected microtubule network (Yadav and Linstedt, 2011). However, Golgi-localised signalling events that regulate the structure and assembly of the Golgi stacks also play a key role in mediating Golgi reorientation, in addition to the microtubule network (Yadav and Linstedt, 2011). GCKIII kinases are likely to be key regulators of such localised signalling events (Preisinger et al., 2004; Fidalgo et al., 2010). Crucially, of the three GCKIII kinases, we found that only MST3 and MST4 interacted with FAM65A. The third GCKIII kinase, YSK1, was not found to be active in our system (Fig. 3A,B). This is in line with previous studies that have demonstrated YSK1 to be specifically activated in response to oxidative stress (Zhou et al., 2009). Furthermore, in agreement with our data that MST3 and MST4 locally act to inhibit Golgi reorientation, MST4 has been previously shown to negatively regulate directional migration, whereas YSK1 has been shown to be a positive regulator of Golgi reorientation and directional migration (Preisinger et al., 2004). Although this difference could be due to YSK1 and MST4 having different Golgi-specific substrates (Preisinger et al., 2004), our results suggest that the opposing effects could also be due to differential association with FAM65A.

GCKIII kinases, along with their interacting protein CCM3, are known to be constituents of a conserved multi-protein complex known as the striatin-interacting phosphatase and kinase (STRIPAK) complex (Goudreault et al., 2009). In addition to GCKIII kinases and CCM3, STRIPAK contains the catalytic and scaffolding subunits of protein phosphatase-2A (PP2A) along with a specific family of regulatory subunits known as striatins (STRN1, STRN3 and STRN4), as well as STR-interacting proteins STRIP1 and STRIP2, Mob-domain-containing protein MOB3 and the membrane-anchoring protein SLMAP (Goudreault et al., 2009; Kean et al., 2011). The STRIPAK complex has been shown to negatively regulate MST3 and MST4 kinase activities, most likely by dephosphorylating their activation segments through PP2A (Madsen et al., 2015). Notably, we did not detect striatins, MOB3, SLAMP or STRIP1 and STRIP2 amongst FAM65A-intercating proteins, either by mass spectrometry (Table S2) or immunoblotting (data not shown) analyses. Instead, two other PP2A regulatory subunits (PPP2R2A and PPP2R5E) along with the catalytic and scaffolding PP2A subunits were identified amongst the FAM65A-interacting proteins (Table S2). These results indicate that although a specific PP2A complex is likely to be associated with FAM65A, this complex is distinct from STRIPAK as it lacks several key STRIPAK components. The functional relevance of the FAM65A-associated PP2A complex remains to be determined. We also identified several YWHA/14-3-3 proteins that interact with FAM65A in a RHO-dependent manner. YSK1, but not MST4, has been shown to phosphorylate YWHAZ/14-3-3ζ on Ser58, and this has been suggested to be important for the regulation of cell polarity and directional migration (Preisinger et al., 2004). We did not detect any change in YWHAZ/14-3-3ζ phosphorylation in response to RHO, FAM65A or MST3 and MST4 loss (data not shown), suggesting that YWHA/14-3-3 proteins are unlikely to be substrates of the FAM65A-associated MSTs. It remains to be determined whether YWHA/14-3-3 association with FAM65A is functionally relevant to the regulation of Golgi reorientation.

FAM65A has two orthologues in humans named FAM65B and FAM65C. Nothing is known about FAM65C, but FAM65B has also been recently shown to interact with RHOA (Rougerie et al., 2013). However, FAM65A and FAM65B only share 47% protein sequence similarity, and accordingly, there seems to be several functional differences between the two orthologues. First, although FAM65A only associates with active RHO proteins, interaction of FAM65B with RHOA seems to be constitutive (Rougerie et al., 2013; Gao et al., 2015). Second, FAM65A and FAM65B seem to localise differently within the cell, with FAM65B being primarily localised to the plasma membrane (Diaz-Horta et al., 2014; Gao et al., 2015), as opposed to the Golgi and cytoplasmic punctae containing FAM65A. Nevertheless, we have observed that loss of FAM65A can trigger RHO hyper-activation (Fig. S4A), and crucially, the main function ascribed to FAM65B is to act as an inhibitor of RHOA activity (Rougerie et al., 2013; Gao et al., 2015). Moreover, we have observed that depletion of CCM3 and MSTs can also result in RHO hyperactivation (Fig. S4B), although the mechanism by which this hyperactivation occurs is unclear at the moment. It remains to be determined whether FAM65B can also interact with CCM3 and MSTs, and if so, whether the negative regulation of RHO activity reported for FAM65B is dependent on these interactions. As FAM65B was not expressed in our cells, we could not assess the functional similarities and differences between the two orthologues in this study.

Mutations of the *CCM3* gene have been linked to cerebral cavernous malformations – vascular abnormalities characterised by dilated leaky cerebral lesions that can lead to brain haemorrhage (Draheim et al., 2014). The exact mechanism by which cerebral cavernous malformations arise is still subject to debate, with deregulation of several signalling pathways such as RHO (Richardson et al., 2013; Stockton et al., 2010; Borikova et al., 2010; Whitehead et al., 2009), TGFβ (Maddaluno et al., 2013), β-catenin (Bravi et al., 2015) and MEKK3–KLF2 or MEKK3–KLF4 (Cuttano et al., 2016; Zhou et al., 2016; Renz et al., 2015) having been demonstrated to be involved in development and progression of the disease. Crucially, loss of the CCM3 interaction with GCKIII kinases seems to be the crucial feature of all disease-associated *CCM3* mutations (Fidalgo et al., 2010). We here reveal that in the context of polarity regulation, CCM3 functions by linking MSTs to FAM65A (Fig. 8E). It remains to be determined whether disruption of the RHO–FAM65A–CCM3–MST pathway could be involved in triggering the formation of cerebral vascular lesions, presumably through an initial defect in cell polarisation. Interestingly, FAM65A provides a link between RHO and CCM3, and hyperactivated RHO signalling in endothelial cells has been shown to be a common feature of cerebral cavernous malformations (Richardson et al., 2013). We speculate that such hyperactivation could be due to disruption of the RHO–FAM65A–CCM3–MST cascade (Fig. S4). Determining whether inhibition of Golgi reorientation downstream of RHO is involved in initiating the formation of vascular lesions in cerebral cavernous malformations, as well as revealing the mechanism through which Golgi-localised MSTs regulate reorientation, could prove to be crucial for devising novel therapeutic approaches against the early molecular events that trigger the disease.

## MATERIALS AND METHODS

### Reagents, antibodies, and plasmids

HeLa cells were authenticated using the LGC Standards Cell-Line Authentication service. TAT-C3 (CT04) was purchased from Cytoskeleton Inc. and used at 2 µg/ml. All siRNAs were purchased from Dharmacon (ON-TARGETplus SMARTpools, unless stated otherwise) and used at 10 nM. Transfections were performed using Thermo Fisher Scientifics' Lipofectamine RNAiMAX (siRNA) and Lipofectamine 2000 (DNA) reagents. Mouse monoclonal antibodies against RHOA (sc-418), RHOB (sc-8048), MST3 (sc-135993), MST4 (sc-376649), CCM3 (sc-365586), Ezrin (sc-58758) and myosin light chain 2 (MYL9, MYL12A and MYL12B) (sc-28329) were purchased from Santa Cruz Biotechnology. Goat polyclonal antibody against YSK1 and MST4 (sc-6865) was also from Santa Cruz Biotechnology. Rabbit polyclonal antibody against FAM65A (HPA005923) was from Sigma. Rabbit monoclonal antibodies against RHOC (3430), phosphorylated myosin light chain 2 (at Thr18 and Ser19) (3674), phosphorylated Ezrin (3726), Myc tag (2276) and GM130 (12480), as well as rabbit polyclonal antibodies against MST3 (3723) and MST4 (3822) were all from Cell Signaling Technology. Mouse monoclonal antibody against AKT (2920) was also from Cell Signaling Technology. Mouse monoclonal anti-GAPDH antibody was from Novus Biologicals. Rabbit polyclonal antibody against 14-3-3 proteins (ab9063) was purchased from Abcam. Rabbit polyclonal antibody against phosphorylated GCKIII proteins (ab76579) was also from Abcam. All secondary antibodies for immunostaining were from Molecular Probes. All secondary antibodies for immunoblotting were from LI-COR Biosciences. The antibody dilutions used for western blotting are default concentrations recommended by the suppliers. The subcellular fractionation kit was purchased from Pierce (78840). FAM65A full ORF Gateway Entry clone (Clone ID: 100062185) was purchased from Open Biosystems. Full-length and truncated GFP–FAM65A mutants were generated by Gateway cloning as described previously (Mardakheh et al., 2010). Myc-tagged constitutively active (Q63L) and dominant negative (T19N) RHOA constructs were a gift from Alan Hall (Sloan-Kettering Institute, NY, USA). The GST–RHOA bacterial expression vector has been previously described (Ridley et al., 1993). CRISPR pSpCas9 (BB)-2A-Puro plasmid (pX459) was obtained from Addgene (plasmid ID 48139). The following 20-mer guide sequences were cloned into the sgRNA site of pX459, as described in Bauer et al. (2015), to generate specific CRISPR plasmids: 5ʹ-GTGTACACGGCGCTGAAGCG-3ʹ (FAM65A), 5ʹ-CAGATAGGATCCATAATATT-3ʹ (MST3) and 5ʹ-TTGGACAGCCACCGGCGAGT-3ʹ (MST4).

### Generation of CRISPR knockout cell lines

HeLa cells were transfected with specific CRISPR plasmids. The next day, the cells were put under Puromycin selection (2 µg/ml) for 24 h, before washing the Puromycin off, trypsinising the cells and seeding them into 96-well tissue culture plates at 50 cells per plate to obtain single-cell clones. Grown out clones were split into two, with half of the cells being seeded on a PerkinElmer CellCarrier 96 plate and screened for successful loss of the target proteins on an Operetta high-content imaging system (PerkinElmer), using immunofluorescence staining against FAM65A, MST3 or MST4. Successful knockout clones were identified based on the low intensity of their immunostaining and subsequently verified by immunoblotting.

### Pulldowns, immunoprecipitations and immunofluorescence

All steps of pulldowns and immunoprecipitations were performed at 4°C. Cells were lysed in a non-ionic lysis buffer (50 mM Tris-HCl pH 7.5, 150 mM NaCl, 1% NP-40, 10 mM MgCl_2_, plus phosphatase and protease inhibitor cocktails from Roche) and cleared by centrifugation at 8000 ***g*** for 20 min. GST pulldowns were performed using purified bacterially expressed GST or GST–RHOA immobilised on glutathione–Sepharose beads (5–7.5 mg/ml bait concentration). 50 µl of the bed volume of beads was added to ∼1.5 ml of lysates (2–2.5 mg/ml lysate concentration) for 1 h, before three 2-ml washes in the lysis buffer, and elution in 50 µl of boiling 2× SDS-PAGE sample buffer. Endogenous FAM65A immunoprecipitation was performed using 10×15-cm dishes of cells that had been transfected with a non-target siRNA or an siRNA against FAM65A. Cells were trypsinised, pelleted and lysed in 1 ml of lysis buffer. After centrifugation, cleared lysates (24 mg/ml) were subjected to immunoprecipitation using 3 µg of anti-FAM65A antibody, immobilised on Sepharose-G beads. After 1 h, the beads were washed five times in 1 ml of lysis buffer and resuspended in 40 µl of boiling 2× SDS-PAGE sample buffer. All GFP and Myc-tag immunoprecipitations were performed using µMACS GFP or Myc Isolation kits from Miltenyi Biotec, according to manufacturer's instructions, with the addition of 10 mM MgCl_2_ to the kit buffers. For immunofluorescence staining, cells were fixed for 10 min in PBS+4% formaldehyde, permeabilised and blocked for 1 h in PBS+5% goat serum+0.1% saponin before being incubated with primary and secondary antibodies for 1 h in PBS+1% BSA+0.1% saponin. Three PBS washes were performed between each step. The anti-GM130 antibody was used at (1:200). Anti-MST4 and anti-FAM65A antibodies were used at 1:50. Secondary antibodies were used at (1:400). Confocal imaging was performed on a Zeiss LSM 710 confocal microscope with a 63× NA oil objective at optimal aperture settings and four-times averaging per image. Colocalisation was quantified by calculating Pearson's correlation between the red and green channels using ImageJ.

### Quantitative proteomics

Each GST pulldown SILAC mix was resolved by performing SDS-PAGE and cut into seven sections. In-gel trypsin digestions, peptide extractions and liquid-chromatography-coupled tandem mass spectrometry (LC-MS/MS) analyses were performed as described previously (Mardakheh et al., 2015). GFP immunoprecipitations were trypsin-digested using Filter Assisted Sample Preparation (FASP) (Wiśniewski et al., 2009), desalted and analysed by performing LC-MS/MS as described previously (Mardakheh et al., 2015). Mass spectrometry search and quantifications were done by using Maxquant (Cox and Mann, 2008), as described previously (Mardakheh et al., 2015). GST–RHOA pulldown data files were searched against the Human UniProt database. GFP–FAM65A immunoprecipitation data files were searched against Human IPI (version 3.68). Mass spectrometry raw data files and search results were deposited in the ProteomeXchange Consortium (Vizcaíno et al., 2014) by using the PRIDE partner repository (accessions PXD004934 and PXD004933). All downstream proteomics data analyses were performed by Perseus software (Tyanova et al., 2016). Significant interacting proteins were identified using one-sided Significance A (SigA) outlier test on the averaged ratios from all runs. A *P*-value cut-off of 0.01 was used for GST–RHOA pulldowns, whereas a *P*-value cut-off of 0.05 was used for GFP immunoprecipitations. Relative stoichiometries of the proteins that interacted with FAM65A were calculated by subtracting their Maxquant-calculated iBAQ values in GFP–FAM65A immunoprecipitations from those of GFP-only immunoprecipitations. The subtracted values were then normalised to the FAM65A-subtracted iBAQ values before being averaged between the two reciprocally labelled experiments. This was performed on two duplicate runs.

### Wound healing assays

For wound healing, 0.5×10^5^ cells were seeded onto the two wells of an iBidi wound healing culture insert (IB-80206) with a defined gap of 500 µm±50 µm in between, and allowed to adhere for 24 h, before serum starvation for another 24 h. The culture insert was then removed with a pair of tweezers, and Dulbecco's modified Eagle's medium with 10% FBS was added to trigger cell migration into the gap. When indicated, TAT-C3 was added 4 h prior to insert removal. For analysis of Golgi reorientation, cells were fixed after 3 h and immunostained with anti-GM130 antibody and DAPI to visualise the Golgi and the nucleus, respectively. Transmission light microscopy was used to visualise the cells. The direction of the Golgi apparatus was defined by drawing a vector from the centre of nucleus to the Golgi. For analysis of wound closure, cells were subjected to time-lapse microscopy in a live-imaging chamber (37°C, 10% CO_2_) after removal of the insert, using a 10× NA objective on a Nikon Eclipse TE2000-S inverted epifluorescence microscope. The distance migrated by each wound edge after 24 h was quantified by using ImageJ.
